# Repurposing of a clinically used anti-HPV agent to prevent and treat SARS-CoV-2 infection as an intranasal formulation

**DOI:** 10.1038/s41392-021-00737-7

**Published:** 2021-08-26

**Authors:** Chen Hua, Qinhai Ma, Yun Zhu, Shuai Xia, Zezhong Liu, Lin Li, Lu Lu, Nanshan Zhong, Shuwen Liu, Zifeng Yang, Shibo Jiang

**Affiliations:** 1 Key Laboratory of Medical Molecular Virology (MOE/NHC/CAMS), School of Basic Medical Sciences, Shanghai Institute of Infectious Disease and Biosecurity, Fudan University, Shanghai, China; 2 State Key Laboratory of Respiratory Disease, National Clinical Research Center for Respiratory Disease, Guangzhou Institute of Respiratory Health, the First Affiliated Hospital of Guangzhou Medical University, Guangzhou, China; 3 National Laboratory of Biomacromolecules, Institute of Biophysics, Chinese Academy of Sciences, Beijing, China; 4 State Key Laboratory of Organ Failure Research, Guangdong Provincial Key Laboratory of New Drug Screening, School of Pharmaceutical Sciences, Southern Medical University, Guangzhou, China; 5 Guangzhou Laboratory, Bio-Island, Guangzhou, China

**Keywords:** Drug development, Target validation

**Dear Editor,**

Fan et al. have shown that whey protein, such as that found in human, bovine, and goat milk, could effectively inhibit SARS-CoV-2 infection, but they did not identify the components in whey protein having this activity.^1^ Since β-lactoglobulin (β-LG) is the main component of whey protein, we herein tested the anti-SARS-CoV-2 activity of β-LG and 3-hydroxyphthalic anhydride (3HP)-modified β-LG (3HP-β-LG), an active component in anti-human papillomavirus (HPV) biological dressing (JB01-BD) that has been used in clinics since 2013 to block cervical infection of HPV.^2–4^ Surprisingly, β-LG could not inhibit SARS-CoV-2 pseudovirus (PsV) infection in 293T/ACE2 cells at the concentration as high as 40 µM. In contrast, 3HP-β-LG, like Griffithsin (GRFT) that targets the SARS-CoV-2 S-RBD and EK1 that targets the SARS-CoV-2 S-HR1, was very effective in inhibiting SARS-CoV-2 PsV infection with a half-maximal inhibitory concentration (IC_50_) of 0.84 ± 0.29 µM (Fig. 1a). Similarly, 3HP-β-LG could also inhibit SARS-CoV-2 PsV infection in Calu-3 cells, a lung epithelial cell line (Supplementary Fig. S1). We found that 3HP-β-LG could also inhibit authentic SARS-CoV-2 infection in Vero-E6 cells with an IC_50_ of 2.31 μM, while, again, β-LG had no inhibitory activity at the concentration up to 40 µM (Fig. 1b).

**Fig. 1:**
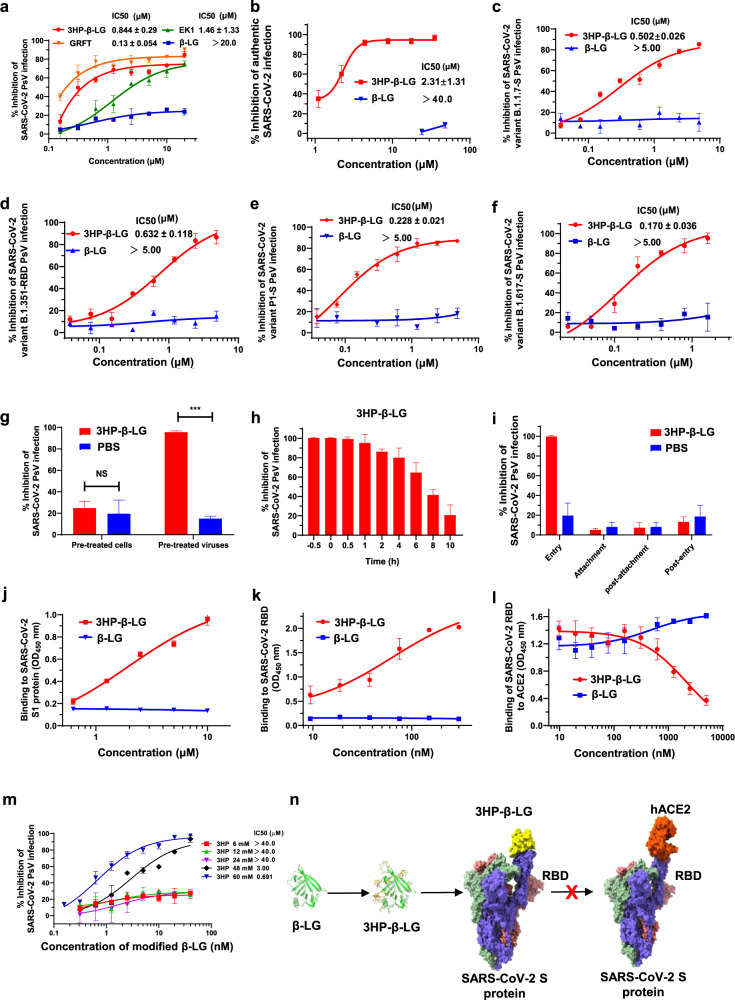
Inhibitory activity of 3HP-β-LG on infection by the pseudotyped (**a**) and authentic (**b**) SARS-CoV-2, as well as the pseudotyped SARS-CoV-2 variant B.1.1.7 (**c**), variant B.1.351 (**d**), variant P1 (**e**), and variant B.1.617.1 (**f**) with mutations in S protein or RBD. Experiments were repeated twice, and the data are expressed as means ± SD (error bar). **g** Cell- and virus-washout assays. For cell-washout assay, 293T/ACE2 cells were incubated with 3HP-β-LG at 37 °C for 1 h and washed with DMEM via centrifuge to remove the unbound 3HP-β -LG. This was followed by the addition of SARS-CoV-2 PsV and detection of infectivity. For virus-washout assay, SARS-CoV-2 PsV was incubated with 3HP-β-LG at 37 °C for 1 h and washed with DMEM via ultrafiltration using Amicon Ultra-100 filters (Millipore) to remove unbound 3HP-β-LG. This was followed by the addition of 293T/ACE2 cells and detection of SARS-CoV-2 PsV infectivity. Data points represent means ± s.e.m. from triplicate samples. NS not significant. **h** Time-of-addition assay. The inhibitory activity of 3HP-β-LG (10 μM) at 0.5 h before adding SARS-CoV-2 PsV (−0.5 h) and at 0, 0.5, 1, 2, 4, 6, and 8 h after adding SARS-CoV-2 PsV was compared. Data points represent means ± s.e.m. from triplicate samples. **i** A series of assays were performed to determine which step of the viral entry process was blocked. Binding of 3HP-β-LG and β-LG to SARS-CoV-2 S1 protein (**j**) and its RBD (**k**) was detected with ELISA. Each sample was tested in triplicate, and the experiment was repeated twice. **l** Inhibition of 3HP-β-LG and β-LG on binding between SARS-CoV-2 S protein RBD and ACE2 was detected with ELISA. Each sample was tested in triplicate, and the experiment was repeated twice. **m** Inhibitory activity of 3HP-β-LG modified with different concentrations of 3HP on SARS-CoV-2 PsV infection. **n** The proposed mechanism of action of 3HP-β-LG against SARS-CoV-2 infection

Very interestingly, 3HP-β-LG was also effective against infection by pseudotyped SARS-CoV-2 variants with increased transmissibility and reduced sensitivity to SARS-CoV-2 neutralizing antibodies,^5^ such as the variant B.1.1.7 with mutations in S protein that include HV 69-70 deletion, Y144 deletion, N501Y, A570D, D614G, P681H, T716I, S982A, and D1118H (Fig. 1c); the variant B.1.351 with mutations in S protein RBD that include K417N, E484K, and N501Y, with IC_50_ ranging from 0.2 to 0.6 μM (Fig. 1d); the variant P1 with mutations in S protein that include L18F, T20N, P26S, D138Y, R190S, K417T, E484K, N501Y, D614G, H655Y, T1027I, and V1176F (Fig. 1e); and the variant B.1.617.1 (version: 2021-04-21, EPI_ISL_1704581) with mutations in S protein that include D614G, E484Q, H1101D, L452R, P681R, Q1071H, the IC50 value is 0.170 ± 0.036 µM (Fig. 1f). β-LG, however, exhibited no inhibitory activity against PsV infection by any SARS-CoV-2 mutant at the concentration at 5 μM (Fig. 1c–f).

To determine whether its inhibitory activity is related to its cytotoxicity, we performed the CCK8 assay. As shown in Supplementary Fig. S2, 3HP-β-LG exhibited no cytotoxicity to Vero-E6 cells or 293T/ACE2 cells at the concentration up to 80 µM, suggesting that 3HP-β-LG has a good in vitro safety profile consistent with our previous report.^2^

Next, we carried out a series experiments to elucidate the mechanism of action of 3HP-β-LG against SARS-CoV-2 infection. First, we performed cell- and virus-washout assays to determine whether 3HP-β-LG inhibits SARS-CoV-2 infection by acting on target cells or virus. 3HP-β-LG was preincubated with 293T/ACE2 cells at 37 °C for 1 h, followed by washing cells with DMEM via centrifuge before adding SARS-CoV-2 PsV. No significant inhibitory effect was detected. On the contrary, when 3HP-β-LG was incubated with SARS-CoV-2 PsV at 37 °C for 1 h, followed by washing the virions with DMEM via ultrafiltration to remove the unbound proteins, the treated SARS-CoV-2 PsV failed to infect 293T/ACE2 cells, indicating that 3HP-β-LG inhibits SARS-CoV-2 infection by interacting with the virus, not the host cells (Fig. 1g). Similarly, the result from an immunofluorescence assay has shown that Fc-tagged SARS-CoV-2 S1 protein could bind, via its RBD, to ACE2-expressing 293T (293T/ACE2) cells, but not to 293T cells without ACE2. Unlike β-LG, 3HP-β-LG could effectively block the interaction between SARS-CoV-2 S1 protein and ACE2 receptor on 293T/ACE2 cells (Supplementary Fig. S3). Second, using a time-of-addition assay, we found 3HP-β-LG (40 µM) able to fully block SARS-CoV-2 PsV infection when it was added to the cells at −0.5, 0, 0.5, and 1 h post infection, but its inhibitory activity gradually decreased from 2 to 10 h post infection (Fig. 1h), suggesting that 3HP-β-LG inhibits SARS-CoV-2 infection by blocking virus entry into the host cells. Third, a series of experiments (Supplementary Fig. S4) was performed to determine the precise stage of viral entry, and results indicate that 3HP-β-LG acts at the early virus entry stage, rather than the attachment, post-attachment, or post-entry stage (Fig. 1i). Fourth, using an enzyme-linked immunosorbent assay (ELISA) and Biolayer Interferometry (BLI, OctetRED96), we found that 3HP-β-LG could bind to the recombinant SARS-CoV-2 S1 (Fig. 1j and supplementary Fig. S5a) and SARS-COV-2 S protein RBD (Fig. 1k and Supplementary Fig. S5b), respectively, in a dose-dependent manner, while β-LG had no apparent binding at the concentration up to 10 µM and 300 nM, respectively. Furthermore, we found that the 3HP-β-LG could block the binding of RBD to the soluble ACE2 receptor (Fig. 1l).

It has been previously shown that different modification ratio of 3HP can affect the inhibitory activity of the modified proteins.^2^ Therefore, in this study, we evaluated the effect of anhydride concentration, as a modifier of 3HP ratio, on the inhibitory activity of 3HP-β-LG against SARS-CoV-2 PsV infection. Different modification ratios of β-LG were monitored by SDS-PAGE (Supplementary Fig. S6) such that gel band migration was correlated with protein net charges after anhydride modification. When modification ratio increased, we saw that the inhibitory activity of 3HP-β-LG increased accordingly (Fig. 1m and Supplementary Fig. S7), suggesting that the inhibitory activity of 3HP-β-LG on SARS-CoV-2 PsV infection is correlated with the ratio of chemical modification and the number of net negative charges on its surface, in consistence with our previous study on the anti-HPV activity of 3HP-β-LG.^2^

The net negative charges on β-LG were largely increased after modification with 3HP on the protein surface of 3HP-β-LG (Supplementary Fig. S8). Using computational simulation by AutoDock, we found that 3HP-β-LG protein could be docked onto the RBD of SARS-CoV-2 spike protein. Compared to the reported SARS-CoV-2 complexed with human ACE2 receptor (hACE2), 3HP-β-LG may share a binding site in common with hACE2 on the RBD of SARS-CoV-2 spike protein (supplementary Fig. S9). This means that the binding between 3HP-β-LG and RBD competitively blocks the interaction between RBD and ACE2 receptor, which is in agreement with the above ELISA results (Fig. 1n).

Given that 3HP-β-LG-containing anti-HPV biological dressing has been safely used via intravaginal application to block HPV infection for more than 7 years,^3^ 3HP-β-LG is expected to be safe via intranasal application for prevention and treatment of SARS-CoV-2 infection. Since β-LG is the major component of bovine whey protein, its abundant source and high thermostability^2^ would make 3HP-β-LG an inexpensive and convenient anti-SARS-CoV-2 agent for use anywhere in the world.

## Supplementary information

Supplementary Material

## Data Availability

All the data used for the current study are available from the corresponding author upon reasonable request.

## References

[1] Fan H (2020). The effect of whey protein on viral infection and replication of sars-cov-2 and pangolin coronavirus in vitro. Signal Transduct. Target Ther..

[2] Lu L, Yang X, Li Y, Jiang S (2013). Chemically modified bovine beta-lactoglobulin inhibits human papillomavirus infection. Microbes Infect..

[3] Guo X (2016). A randomized open-label clinical trial of an anti-hpv biological dressing (jb01-bd) administered intravaginally to treat high-risk hpv infection. Microbes Infect..

[4] Hua C (2019). The underlying mechanism of 3-hydroxyphthalic anhydride-modified bovine beta-lactoglobulin to block human papillomavirus entry into the host cell. Front. Microbiol..

[5] Wang P (2021). Antibody resistance of sars-cov-2 variants b.1.351 and b.1.1.7. Nature.

