# Supplementary material for: Repurposing of a clinically used anti-HPV agent to prevent and treat SARS-CoV-2 infection as an intranasal formulation
Source: Signal Transduct Target Ther. 2021 Aug 26;6:318. doi: 10.1038/s41392-021-00737-7 (PMC8390030; doi:10.1038/s41392-021-00737-7)
Supplement: Supplementary file 1 — Supplementary Material [file 41392_2021_737_MOESM1_ESM.docx]

**Supplementary Material for**

**Repurposing of a clinically used anti-HPV agent to prevent and treat SARS-CoV-2 infection as an intranasal formulation**

Chen Hua^1,#^, Qinhai Ma^2,#^, Yun Zhu^3,#^, Shuai Xia^1^, Zezhong Liu^1^, Lin Li^4^, Lu Lu^1^, Nanshan Zhong^2,5^, Shuwen Liu^4,*^, Zifeng Yang^2,5,*^, and Shibo Jiang^1,*^

*Correspondence to: Shuwen Liu (liusw@smu.edu.cn), Zifeng Yang (jeffyah@163.com), Shibo Jiang (shibojiang@fudan.edu.cn)

This PDF file includes:

Materials and Methods

Figures. S1 to S9

Materials and Methods

*Cell lines, viruses and plasmids*

The human primary embryonic kidney cell line (293T) (CRL-3216™) was obtained from the American Type Culture Collection (ATCC). Human hepatoma Huh-7 cells were from the Cell Bank of the Chinese Academy of Sciences (Shanghai, China). SARS-CoV-2 strain (Genebank accession no. MT123290.1) was isolated from COVID-19 patients in Guangzhou, China. The envelope-expressing plasmids of SARS-2-S (pcDNA3.1-SARS-2-S), SARS-S (pcDNA3.1-SARS-S), and the luciferase reporter vector (pNL4-3.Luc.R-E-) were maintained in our laboratory.

*Chemical modification and characterization of 3HP-modified β-LG*

The bovine whey protein β-LG was modified with 3HP as previously described^1,2,3^. Briefly, anhydride 3HP was dissolved in DMSO at a concentration of 1 M. β-LG was dissolved by adding 0.1 M phosphate buffer (pH 8.5), and the final concentration was 20 mg/ml. 3HP was added to the protein solution, and then 5 M NaOH were used to adjust the pH to 9.0, followed by incubation at room temperature for 20 min. This procedure was repeated 4 times to make a final concentration of 60 mM 3HP in the protein solution. After that, the modified β-LG was dialyzed against PBS. To obtain a series of modified β-LG with different anhydride 3HP ratios, β-LG was reacted with 0, 2.5, 5, 12, 25, 36, 48, or 60 mM of HP, respectively. The concentration of 3HP-modified β-LG was quantified by BCA Protein Assay Kit (Takara). Sodium dodecyl sulfate-polyacrylamide gel electrophoresis (SDS-PAGE) was used to visualize the 3HP- modified β-LG.

*Inhibition of pseudotyped SARS-CoV-2 infection in 293T/ACE2 and Calu-3 cells*

SARS-CoV-2 PsV was produced by co-transfecting plasmid pcDNA3.1-SARS-CoV-2-S (encoding for SARS-CoV-2 S protein) and pNL4-3.Luc.R-E- as described previously^4^. Pseudovirus in supernatant was collected 72 h after transfection. The pseudovirus was quantitated as the level of the lenti-p24 antigen by ELISA as described previously^5^. To detect the inhibitory activity of 3HP-β-LG on SARS-CoV-2 PsV infection, 293T/ACE2 or Calu-3 cells were plated at a density of 10^4^ cells per well in a 96-well plate one day prior to infection. PsV was mixed with an equal volume of 3HP-β-LG which was series diluted with PBS at 37 °C for 30 min. The mixture was transferred to the cells. Medium was changed after 12 h, and incubation continued for 48 h. Luciferase activity was analyzed by the Luciferase Assay System (Promega, Madison, WI, USA).

*Inhibition of authentic SARS-CoV-2 replication*

The inhibition assay for authentic SARS-CoV-2 was performed in a biosafety level 3 (BSL3) facility at the Wuhan Research Institute and Beijing Key Laboratory for Animal Models of Emerging and Re-emerging Infectious Diseases, respectively. Inhibition activity of 3HP-β-LG on SARS-CoV-2 was determined by plaque reduction assay^6^. 3HP-β-LG at the indicated concentration was mixed with SARS-CoV-2 (100 TCID_50_) for 30 min and then added to monolayer Vero-E6 cells. After adsorption at 37 °C, the supernatant was removed, and 0.9% methyl cellulose was overlaid on the cells. After 72 h, the plates were fixed and stained. Plaques were counted by fixing with 4% paraformaldehyde and staining with 0.1% crystal violet.

*Cytotoxicity assay*

The cytotoxicity of 3HP-β-LG to HuH-7, Vero-E6, and 293T/ACE2 cells was measured following instructions in the manual provided with the Cell Counting Kit-8 (CCK-8) ^2^. Briefly, serially diluted 3HP-β-LG was mixed with 10,000 cells in each well of a 96-well plate. After incubation at 37 °C for 24 h, the supernatants were replaced by CCK-8 solution. After reaction for 2 h, the absorbance at 450 nm was detected using a microplate reader (Infinite M200PRO, Tecan, USA).

*Time-of-addition assay*

293T/ACE2 cells were plated in a 96-well plate. SARS-CoV-2 PsV was added and then 5 μM 3HP-β-LG at 0, 0.5, 1, 2, 4, 6, 8, and 10 h, followed by incubation at 37°C for 16 h. After replacement of culture supernatant with fresh medium and incubation for an additional 72 h, cells were lysed to determine the entry inhibition ratio, according to the manufacturer's manual (Promega).

*Elucidating the mechanism of action of 3HP-β-LG against SARS-CoV-2 infection*

For the cell-washout assay, 3HP-β-LG (control: PBS) was added to 293T/ACE2 cells and incubated at 37 °C for 1 h. Afterward, the unbound 3HP-β-LG was removed by washing cells with DMEM, followed by adding SARS-CoV-2 PsV and measuring the % inhibition of its infection as described above. For the virus-washout assay, 3HP-β-LG (control: PBS) was incubated with SARS-CoV-2 PsV at 37 °C for 1 h. Then, after SARS-CoV-2 PsV virions were washed with DMEM using ultrafiltration, they were added to 293T/ACE2 cells. The % inhibition of SARS-CoV-2 PsV infection was determined as described above. For the viral entry assay, the mixture of SARS-CoV-2 PsV and 3HP-β-LG (control: PBS) was added to 293T/ACE2 cells for incubation of 1 h at 37 °C and then washed with cold PBS three times. The protocol of viral attachment was the same as that of the viral entry assay, except the mixture was incubated with 293T/ACE2 cells at 4 °C for 1 h. For the post-attachment assay, 293T/ACE2 cells were infected with SARS-CoV-2 PsV at 4 °C for 1 h. After that, virions were removed by washing with cold PBS, followed by adding 3HP-β-LG to the cells at 37 °C. After 1h incubation, 3HP-β-LG was removed. The protocol of post-entry assay was similar to that of the post-attachment assay, except 293T/ACE2 cells were first infected with SARS-CoV-2 PsV at 37 °C for 1 h. The % inhibition of SARS-CoV-2 PsV infection for each experiment was detected as described above.

*Enzyme-linked immunosorbent assay (ELISA)*

3HP-β-LG diluted in PBS was coated onto the wells of a 96-well polystyrene plate (Corning, USA) at 4°C overnight. After blocking with protein-free blocking buffer (Thermo Fisher Scientific, USA), SARS-CoV-2 S (Fc-tag) protein (1 µg/ml) or SARS-CoV-2 RBD (Fc-tag) (10ng/mL) was added to the wells (50 µl/well), and the plate was incubated at 37 °C for 1 h. After washing with PBST three times, mouse anti-Fc-tag-HRP antibody (Abcam, UK) was added at a 1: 1,000 dilution. After incubation at 37 °C for 1 h, wells were washed, and tetramethylbenzidine (Sigma, USA) was added, followed by the measurement of the absorbance at 450 nm.

To test the inhibitory activity of 3HP-β-LG on binding of RBD to human ACE2, a 96-well polystyrene plate was coated with ACE2 protein (2 μg/mL) and blocked with 2% gelatin for 2 h. After 3 washes with PBST, the plate was incubated with RBD (10 ng/mL) in the presence or absence of 3HP-β-LG before measurement of A450 as described above.

*Immunofluorescence assay*

A total of 5×10^5^/mL 293T/ACE2 cells and 293T cells were seeded into 24-well plate 200μl/well and cultured for 24 h. 293T/ACE2 cells and 293T cells were fixed with PBS containing 4% paraformaldehyde for 10 min. After washed with PBST for 3 times, cells were blocked with PBST containing 10% Normal Goat Serum (36119ES03) for 1 h. Human IgG Fc-tagged SARS-CoV-2 S1 protein (SARS-CoV-2-S1-Fc, #COV-VM5S1) (5 μg/mL) were incubated with 3HP-β-LG, β-LG or PBS for 1 h before being added into 293T/ACE2 cells, while 293T cells treated with SARS-CoV-2 Spike Protein were as a control. After incubation of 1 h, cells were washed with PBST for 3 times and FITC conjugated anti-human IgG antibody (1:100, Abcam) were added into 24-well plate for 1 h. After washed for 3 times, cells were incubated with DAPI for 5 min. Cells were washed with PBST and then imaged on fluorescence microscope.

*Biolayer Interferometry (BLI)*

BLI was carried out by OctetRED96 device (Pall FortéBio). For the binding kinetics of 3HP-β-LG, β-LG with SARS-CoV-2 S1 or SARS-CoV-2 RBD, the recombinant SARS-CoV-2 Spike Protein (S1 Subunit, FC Tag, #COV-VM5S1) (5μg/mL) and SARS-CoV-2 RBD-his (2μg/mL) was immobilized onto activated anti-FC or anti-his biosensors (Pall FortéBio) and incubated with 2-fold serial dilutions of 3HP-β-LG and β-LG in kinetics buffer (PBS buffer supplemented with 0.02% Tween 20). The experiments included the following steps at 37°C: (1) equilibration (60 s); (2) immobilization of S1 protein onto sensors (100 s); (3) baseline in kinetics buffer (120 s); (4) association of 3HP-β-LG or β-LG for measurement of k on (300-600 s); (5) dissociation of 3HP-β-LG or β-LG for measurement of k off (300-1000 s); and (6) regenerated by injecting 10 mM glycine HCl, pH2.0, 100 μL/min for 18 s. Data were analyzed using ProteOn Manager 3.1 software.

*Structural* *modeling analysis*

The structures of β-LG (PDB entry 1BEB) and SARS-CoV-2 S protein bound with human ACE2 (PDB entry 7A94) were all acquired from the Protein Data Bank for modeling analysis. The predicted model of 3HP-β-LG was generated using COOT (<https://www.ccp4.ac.uk/>). The interaction model between 3HP-β-LG and SARS-2-CoV S protein was predicted with AutoDock (<http://autodock.scripps.edu/>). All structural figures were generated and analyzed using PyMOL (<https://pymol.org/2/>) and ChimeraX (http://www.rbvi.ucsf.edu/chimerax).

*Statistical Analysis*

Student’s unpaired two-tailed *t*-test was used to compare the differences for all experiments. GraphPad Prism version 5.0 was used to perform all statistical analyses. *P<0.05; **P<0.01; ***P<0.001.

**References**

1 Neurath, A. R. *et al.* Blocking of cd4 cell receptors for the human immunodeficiency virus type 1 (hiv-1) by chemically modified bovine milk proteins: Potential for aids prophylaxis. *J Mol Recognit*. **8**, 304-316 (1995).

2 Li, H. *et al.* Chemically modified human serum albumin potently blocks entry of ebola pseudoviruses and viruslike particles. *Antimicrob Agents Chemother*. **61** (2017).

3 Sun, Z. *et al.* Intranasal administration of maleic anhydride-modified human serum albumin for pre-exposure prophylaxis of respiratory syncytial virus infection. *Viruses*. **7**, 798-819 (2015).

4 Xia, S. *et al.* Inhibition of sars-cov-2 (previously 2019-ncov) infection by a highly potent pan-coronavirus fusion inhibitor targeting its spike protein that harbors a high capacity to mediate membrane fusion. *Cell Res*. **30**, 343-355 (2020).

5 Zhu, Y. *et al.* Identification of a gp41 core-binding molecule with homologous sequence of human tnni3k-like protein as a novel human immunodeficiency virus type 1 entry inhibitor. *J Virol*. **84**, 9359-9368 (2010).

6 Wang, M. *et al.* Remdesivir and chloroquine effectively inhibit the recently emerged novel coronavirus (2019-ncov) in vitro. *Cell Res*. **30**, 269-271 (2020).

Supplementary figures


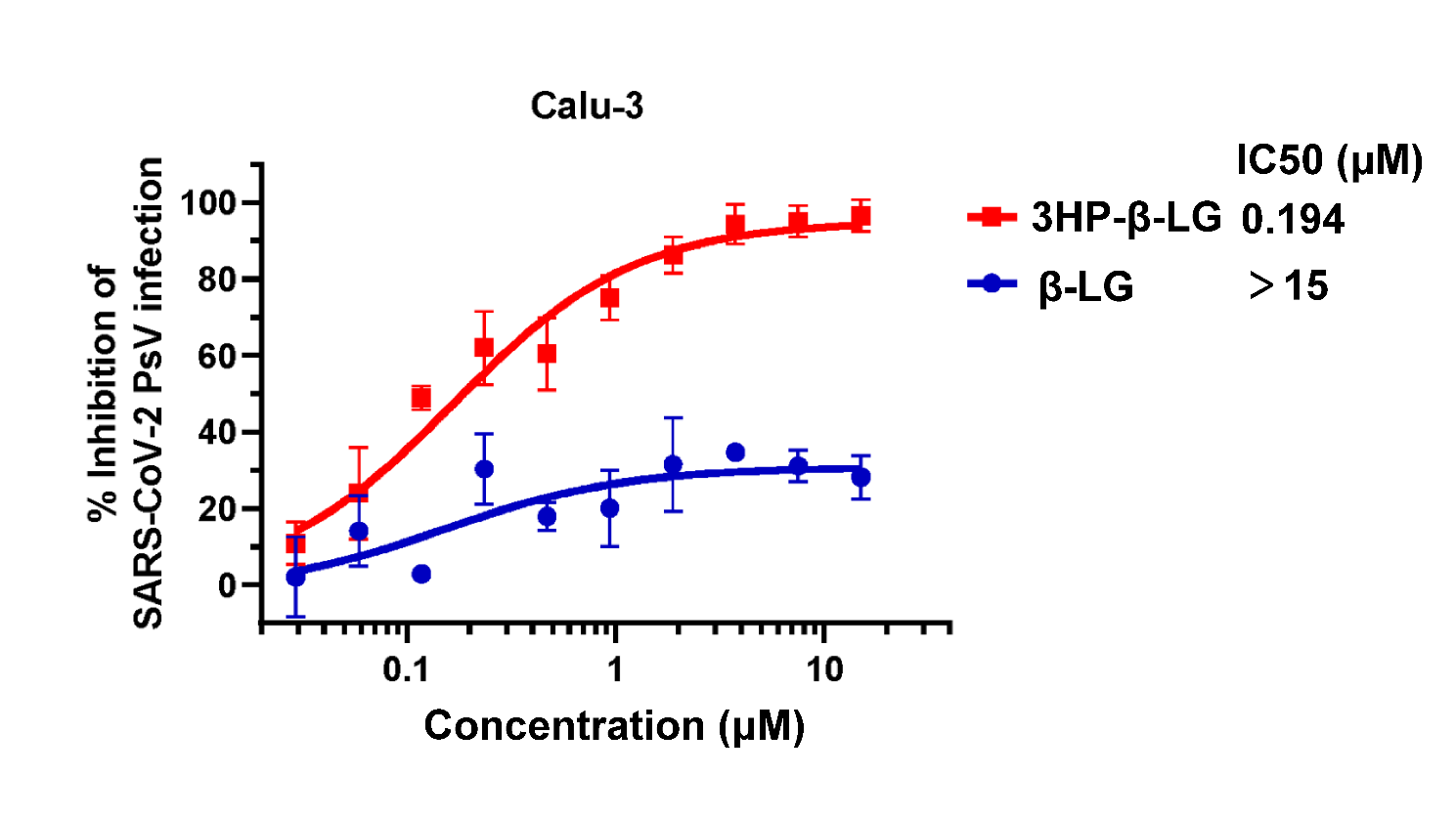


**Figure S1. Inhibitory activity of 3HP-β-LG on infection by the pseudotyped SARS-CoV-2 on Calu-3 cells, a lung epithelial cell line.**

**
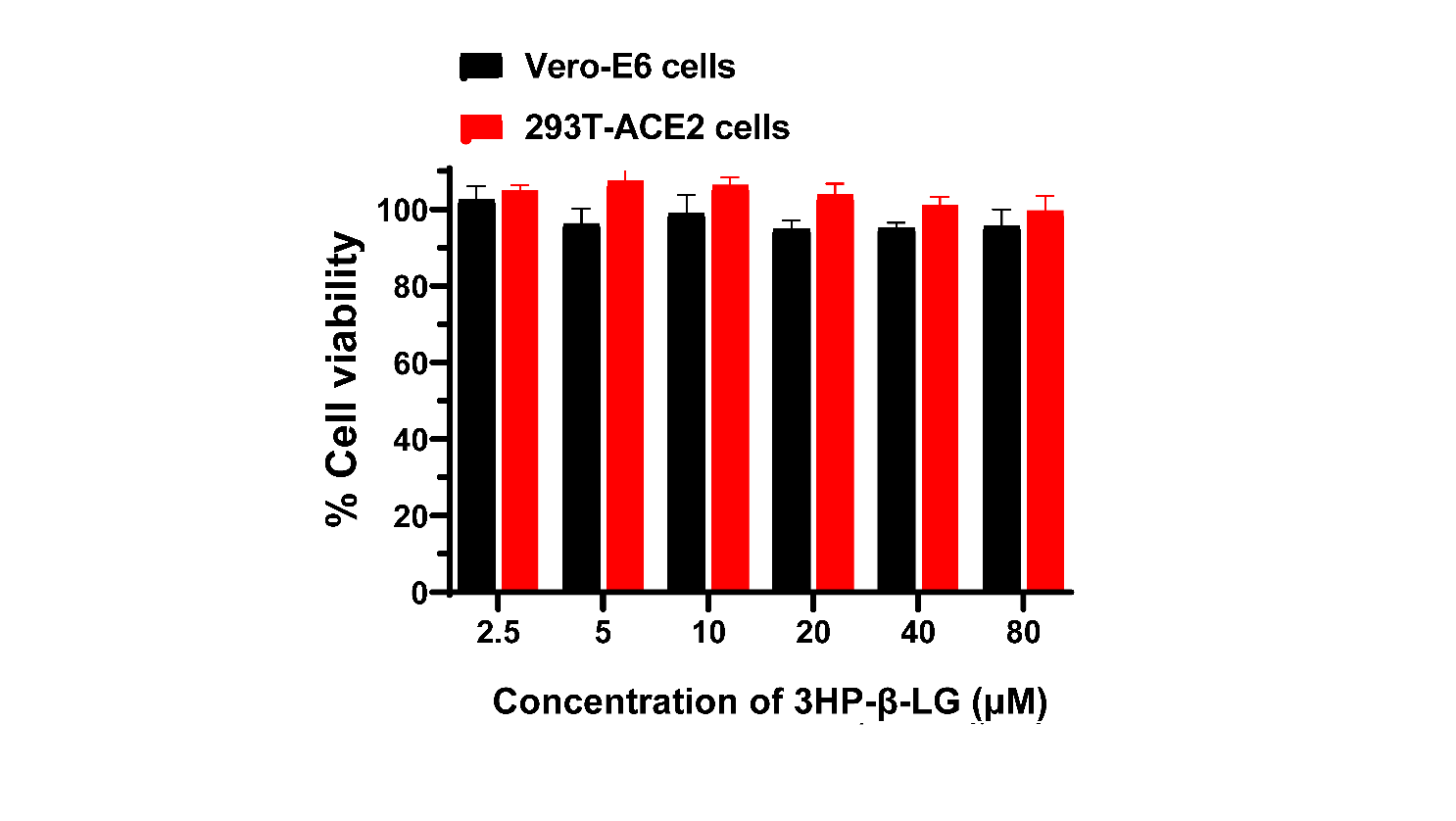
**

**Figure S2. Cytotoxicity of 3HP-β-LG on Vero-E6 and 293T/ACE2 cells as determined by the CCK8 assay.**


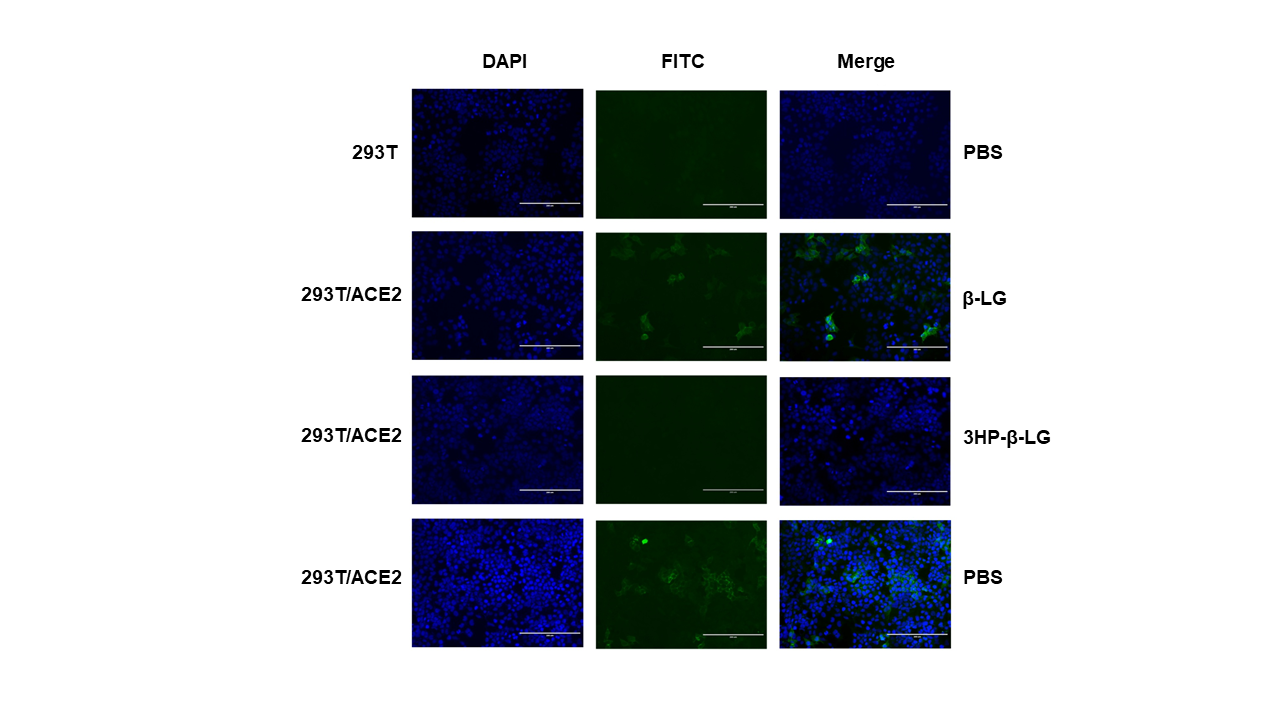


**Figure S3.** **Inhibitory effect of HP-β-LG on the binding of SARS-CoV-2 S protein to ACE2 receptor on 293T/ACE2 cells as determined by using an immunofluorescence assay.**

Human IgG Fc-tagged SARS-CoV-2 S1 protein (SARS-CoV-2-S1-Fc, #COV-VM5S1) (5 μg/mL) was incubated with 3HP-β-LG (10 μM), β-LG (10 μM) or PBS for 1 h before being added into wells containing 293T or 293T/ACE2 cells, followed by addition of FITC-conjugated goat anti-human IgG Fc antibody (5 μg/mL, Abcam #37367). Cell nuclei were stained by DAPI (blue). FITC-stained cells (green) were observed under an immunofluorescent microscope.


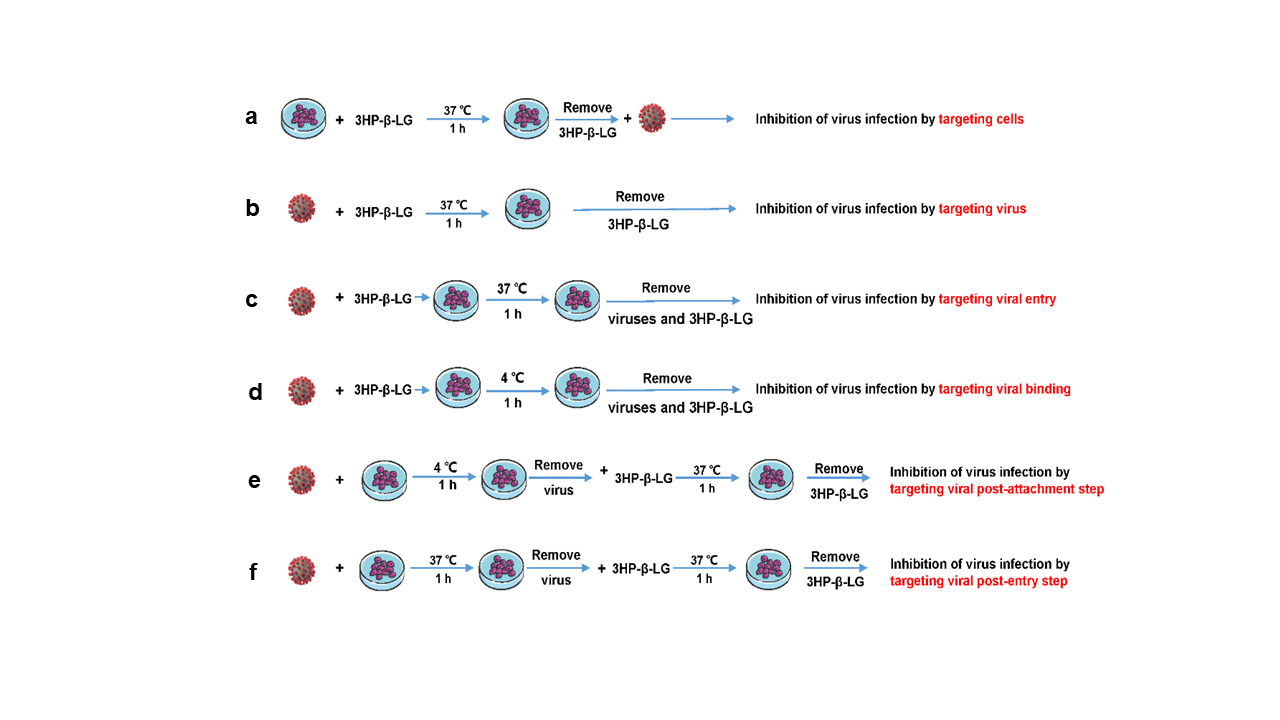


Figure S4. Elucidating the mechanism of action of 3HP-β-LG against SARS-CoV-2 infection.

(a) For the cell-washout assay, 293T/ACE2 cells were incubated with 3HP-β-LG or PBS at 37 °C for 1 h, followed by the removal of unbound 3HP-β-LG by washing cells before adding SARS-CoV-2 PsV. (b) For virus-washout assay, SARS-CoV-2 PsV was incubated with 3HP-β-LG or PBS at 37 °C for 1 h, followed by the removal of unbound 3HP-β-LG by ultra-filtration before adding 293T/ACE2 cells. (c)For the viral entry experiment, 293T/ACE2 cells were infected with SARS-CoV-2 PsV in the presence of 3HP-β-LG (control: PBS) at 37 °C for 1 h. After that, the virus and 3HP-β-LG were removed during a series of three washes with cold PBS. (d) For the viral attachment assay, 293T/ACE2 cells were incubated with SARS-CoV-2 PsV in the presence of 3HP-β-LG (control: PBS) at 4 °C for 1 h, and then the unbound virus and 3HP-β-LG were removed. (e) For the viral post-attachment assay, 293T/ACE2 cells were first infected with SARS-CoV-2 PsV at 4 °C for 1 h and then washed to remove unbound virions, followed by adding 3HP-β-LG (control: PBS) at 37 °C for 1 h. (f) For the viral post-entry assay, SARS-CoV-2 PsV was incubated with 293T/ACE2 cells at 37 °C for 1 h. After washing to remove the unbound virions, cells were treated with 3HP-β-LG (control: PBS) at 37 °C for another 1 h, followed by removing 3HP-β-LG and measuring the infectivity of SARS-CoV-2 PsV.


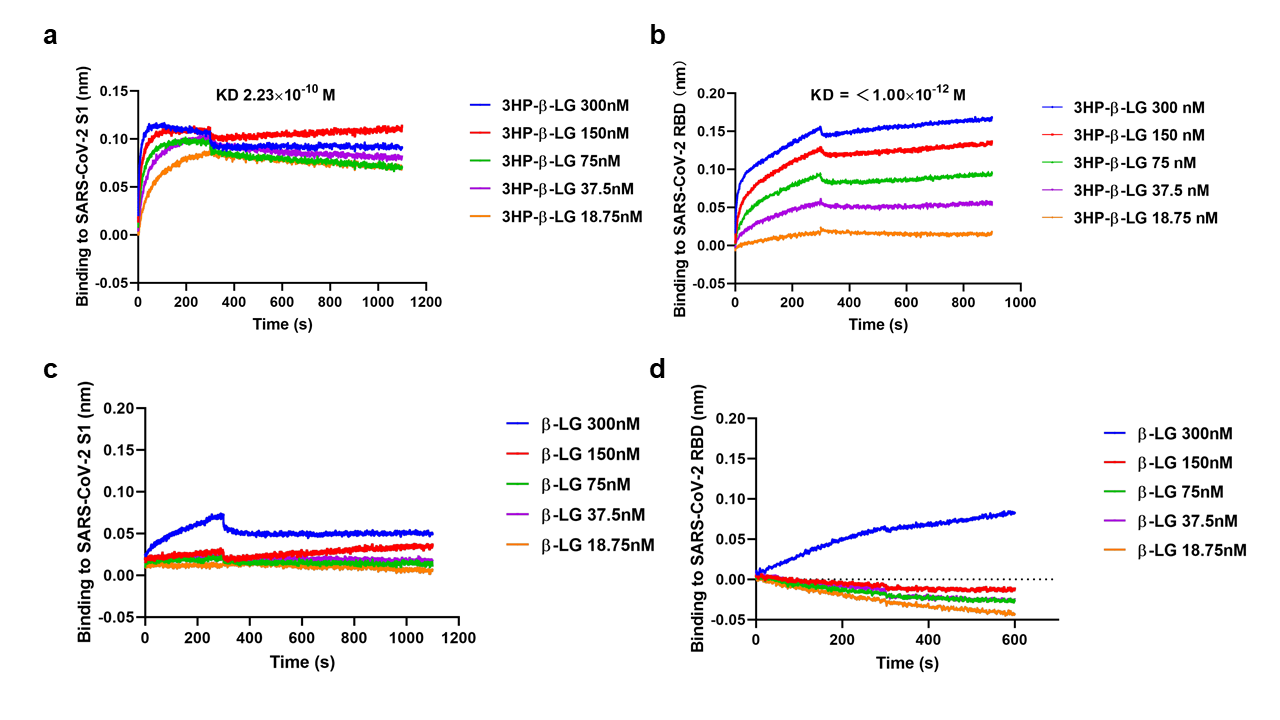


**Figure S5. Binding kinetics of 3HP-β-LG and β-LG to SARS-CoV-2 S protein or RBD.**

Binding kinetics of 3HP-β-LG and β-LG to SARS-CoV-2 S protein (a, c) and SARS-CoV-2 RBD (b, d) as measured by BLI using OctetRED96. Purified SARS-CoV-2 S1-Fc or SARS-CoV-2 RBD-His were immobilized on Anti-hIgG Fc or Anti-his Capture biosensors. The analytes consisted of serial dilutions of 3HP-β-LG or β-LG between 300 to 18.75 nM. Binding kinetics were evaluated using a 1:1 Langmuir binding model by Fortebio Data Analysis 7.0 software.

**
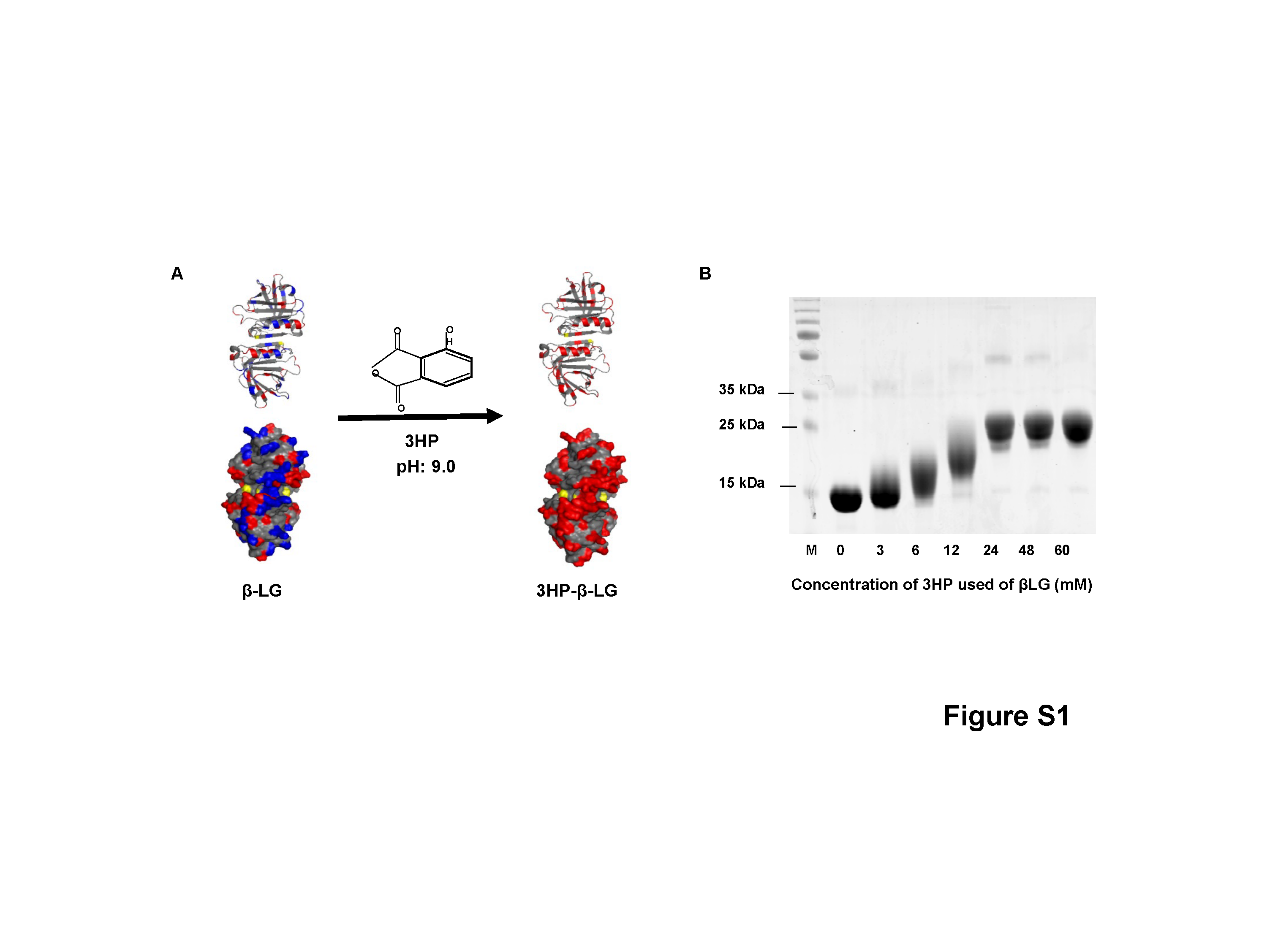
**

**Figure S6. The molecular weight of 3HP-β-LG modified with different concentrations of 3HP was visualized by SDS-PAGE.**


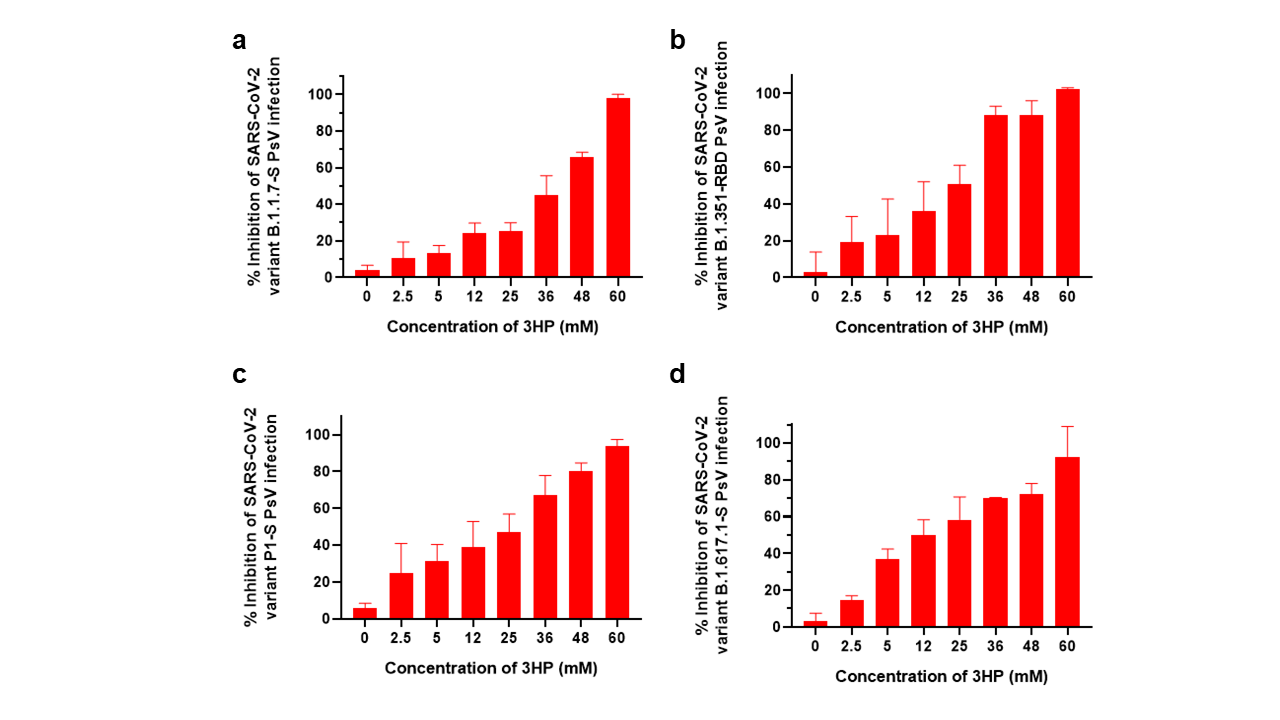


**Figure S7. The effect of anhydride (3HP) concentration for modification of β-LG to generate 3HP-****β-LG against infection by SARS-CoV-2 variants B.1.1.7 (a), B.1.351 (b), P1 (c), and B.1.617.1 (d).**

**Figure S8. The modification of β-LG with 3HP resulting in increase of the net negative charges on** **3HP-β-LG.**


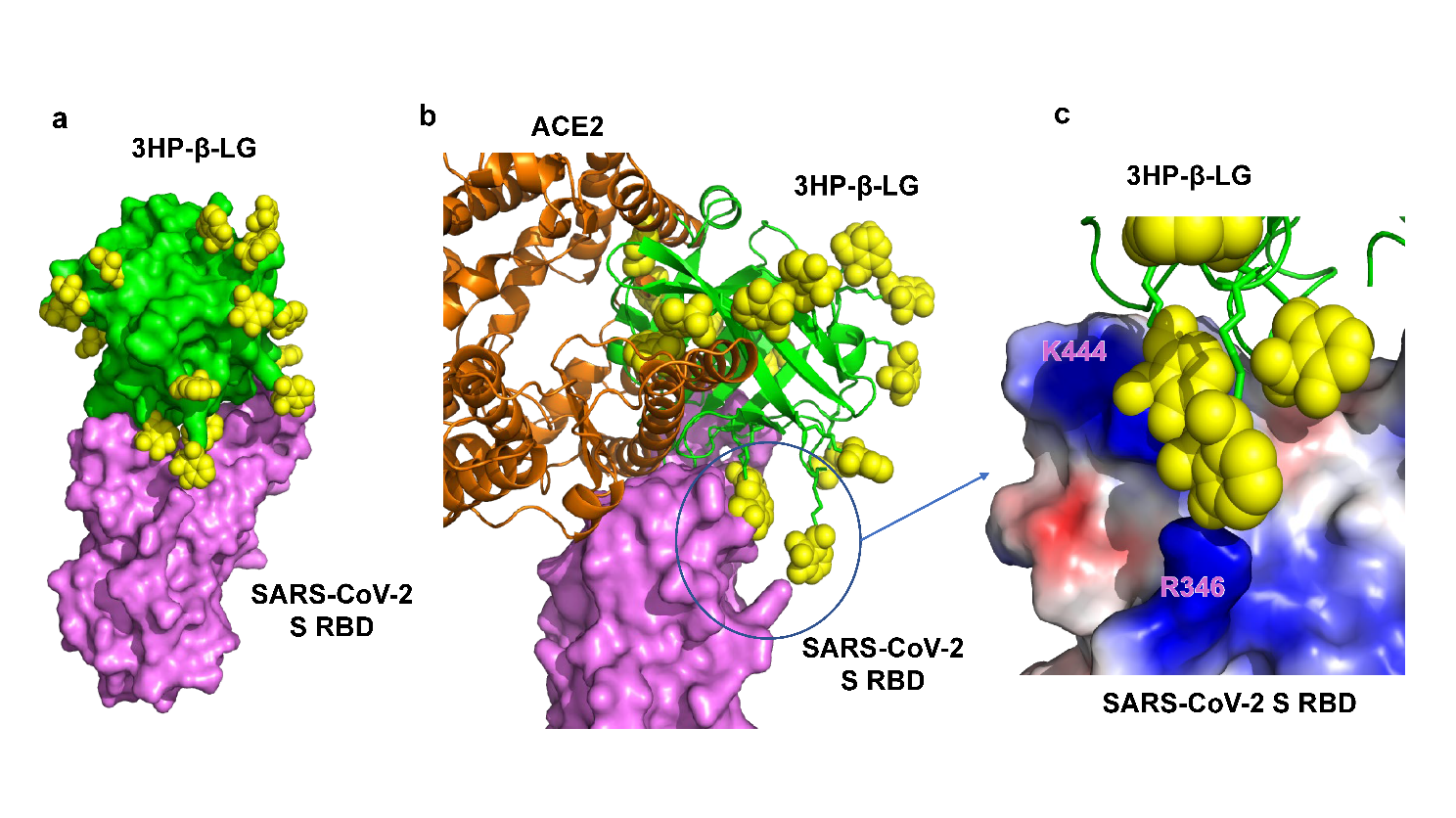
**Figure S9. 3HP-β-LG may interact with the activate site of RBD to disturb ACE2 binding.**

(a) The predicated docking structure of 3HP-β-LG and SARS-CoV-2 spike RBD domain. (b) 3HP-β-LG may share the same interaction site on RBD with ACE2. (c) The 3HP-β-LG may bind to the positively charged regions on RBD.
